# Mobile Dating Applications and the Sexual Self: A Cluster Analysis of Users’ Characteristics

**DOI:** 10.3390/ijerph19031535

**Published:** 2022-01-29

**Authors:** Alexandru Mateizer, Eugen Avram

**Affiliations:** Department of Psychology, Faculty of Psychology and Educational Sciences, University of Bucharest, 050663 Bucharest, Romania; eugen.avram@fpse.unibuc.ro

**Keywords:** sexual self, dating, online sexual activities, cluster analysis, mobile dating applications, sexual satisfaction

## Abstract

The online environment has had a profound sociocultural impact, and its implications pose new challenges to modern-day societies. The digital sexual and dating domains have dramatically affected sexual and romantic behavior and norms, and yet, no empirical studies have addressed the role of the sexual self-concept in driving sexual and romantic online behavior. The aim of this study is to identify reliable sexual self-configurations through a cluster analysis approach and determine whether these profiles are informative in relation to app use motives and sexual outcomes. For this purpose, a total of 244 subjects were recruited. Survey questions measured a set of demographic parameters, motives for app use, online sexual activities, attachment orientations, the sexual self-concept and sexual satisfaction. Five clusters were identified, including two with high levels of sexual drive (“Driven” and “Adventurous”), two with low levels of sexual drive (“Unassured” and “Naïve”) and one with an intermediate level of sexual drive (“Competent”). The clusters differed on gender, attachment styles, app use patterns and sexual characteristics. These findings provide insight into how the sexual self-concept relates to the interaction between individuals and the online sexual and dating scenes.

## 1. Introduction

Digital media has become part of our everyday lives and has had a profound impact on the way identity is formed [1] and on how people organize and approach a wide range of experiences. The practical implications that followed the ever-increasing space of virtual interconnectedness and ease of access to information, services and people are highly complex and pose new challenges to individuals in modern-day societies. One such important feature of the online environment is constituted by social media and dating apps that have offered unprecedented access to the dating market and provided opportunities for interacting and meeting new people. Although research has focused on individual characteristics that drive app use, such as personality traits, attachment or sexual desire [2,3], the way in which people’s app use motives vary as a function of their sexual self-perception has not been previously studied. Although there is interest regarding specific effects of interaction, a comprehensive model of online/offline sexuality has yet to be discovered and tested. A substantial limitation of psychological research in sexuality has been its focus on the effects of separate factors, while possible effects relating to configurations of individual characteristics and the various degrees of interpersonal variability have been mostly overlooked [3,4]. Given the significant impact that the digital domain has on both individuals and the sociocultural landscape, this study aims to further advance research on this topic by investigating characteristics of online app use and online sexual activities within specific configurations of sexual self-characteristics.

The sexual self-concept is considered a dynamic construct that forms by organizing perceptions of personal sexual qualities into a cohesive, internalized structure [5]. Research on this topic has focused on the sexual self-concept as a multidimensional construct dealing with evaluations of cognitive, affective, interpersonal, behavioral, and physiological aspects of sexuality, as well as attributions of desire and arousal [6,7,8,9]. In other words, apart from individual conceptualizations of sex, the sexual self is defined as how people think and feel about themselves as sexual beings, both as sexual individuals alone as well as in the context of a sexual experience with another person. For this study, a broader sexual self-model was chosen. Based on Buzwell and Rosenthal’s [10] model, Deutsch [5] tested a five lower-order factor model (arousal, exploration, anxiety, sexual self-esteem and sexual self-efficacy), which formed a higher-order latent factor of sexual self, applicable to both genders. We first sought to determine if, by using this model, reliable sexual self-subtypes can be identified among individuals, in an attempt to replicate previous findings. For reference, we used Buzwell and Rosenthal’s [10] study, in which five such profiles were identified in a sample of adolescents and labeled sexually “Competent”, “Driven, “Adventurous”, “Naïve” and “Unassured”.

Two areas where sexuality is involved will be explored in this study, namely digital apps that people use for sexual and/or romantic purposes (e.g., Tinder, Bumble, Happen, etc.) and online sexual activities (e.g., watching pornography, sex chat, etc.). Digital tools have dramatically impacted sexual and romantic behavior and norms, with potentially significant sociocultural consequences [11]. Apps like Tinder or Instagram are at the forefront of this digital realm, reflecting two sets of tools used by young people for their romantic or sexual interactions and self-presentation. Research on dating apps has mainly been focused on motives and outcomes of use. For example, studies investigating the reasons for which people use Tinder have found general themes referring to relational goals, like love interest or sex, intrapersonal goals, like self-enhancement and communication disinhibition, or entertainment purposes, like curiosity, thrill or trendiness [12,13]. Recently, there has been interest in the associations between dating app motives and personality-based features, such as attachment style, self-esteem or sexual desire [14], sensation seeking and sexual permissiveness [12] or dark traits [15,16]. In line with this research, this study aims to both replicate and expand previous results by taking into consideration attachment orientations and sexuality factors, such as sexual satisfaction and online sexual activities, in investigating app use within specific sexual self-configuration frameworks. 

Online sexual activities (OSAs) reffers to the use of the internet for any activity that involves sexuality [17]. Out of the many types of OSAs, for the purpose of this study, two categories will be selected, solitary OSAs (e.g., watching pornography) and partnered OSAs (e.g., sex chat) [18]. Thus far, research concerning OSA has focused mainly on harmful outcomes, such as exposure trauma [19], positive attitudes towards uncommitted sex [20,21], risks for decreased social integration [22], abuse or addiction [23] and sexual dissatisfaction [24,25], reflecting interest in the new and uncertain threats related to the ever-expanding digital domain. In contrast, very few studies have investigated less unpleasant outcomes of OSAs or, indeed, the relation between OSAs and the sexual self-concept. For example, some studies [17,26] found that the majority of internet users engage in OSAs recreationally in ways that do not lead to difficulties in their life, with some even reporting benefits to their levels of sexual activity and improvements in sexual communication. In regards to the sexual self-concept, so far, the only model of interaction was proposed by McKenna et al. [27] in a study conducted on a large number of online groups devoted to mainstream forms of cybersex. They found that the online environment provided a secure scene for sexual self-expression, where individuals became more accepting and less ashamed of their sexual desires and fantasies in connecting with others that share or understand those sexual needs. This research aims to further investigate OSAs in the context of app use and sexual self-features.

In brief, the present study is focused on identifying reliable sexual self-profiles and investigating differences in app use and online sexual behaviors among these groups. We expect to replicate findings in previous studies on related topics and to further expand knowledge on sexuality and the digital domain. 

## 2. Materials and Methods

### 2.1. Participants and Procedure

The research was conducted based on a sample of 244 respondents consisting of 149 females and 95 males, with a mean age of 26.92 (SD = 9.32). The majority of respondents reported using Tinder and Instagram apps for romantic and sexual purposes. Recruitment for this study was done through social media platforms, such as Facebook, and participation invitations were sent via e-mail. The questionnaire application process involved the completion of an online form comprised of several sections: information regarding the confidentiality of personal data, informed consent and the measures used in the study. The ethical approval for this study was obtained from the University of Bucharest.

### 2.2. Measures

Demographics. Demographic variables taken into consideration were age, gender, education, sexual orientation, relationship status, number of sexual partners in the previous 12 months, apps used for romantic or sexual purposes, frequency of use, partner searching online and cyber infidelity.

App use motives. Motives for using apps were measured using the Tinder Motivations Scale (TMS) [28], developed to evaluate reasons for Tinder use. The items were modified in order to convey social and dating apps in general. A total of 24 items are grouped into 6 main motivations for dating app use: Love (5 items; α = 0.909; e.g., “to find a romantic relationship”), Casual Sex (4 items; α = 0.877; e.g., “to find someone to have sex with”), Ease of Communication (5 items; α = 0.844; e.g., “online I’m less shy than offline”), Self-Worth Validation (5 items; α = 0.923; e.g., “to improve my self-esteem”), Thrill of Excitement (2 items; α = 0.835; “because it’s exciting”), and Trend (3 items; α = 0.773; e.g., “everyone uses it”). Each item was scored on a 5-point Likert scale (1 = totally disagree; 5 = totally agree). The final scores were obtained by adding the corresponding items.

Online sexual activities. A series of items were developed specifically for this study in order to assess the frequency of engagement in online sexual activities (OSAs) in the previous 6 months. The items were created in accordance with themes presented in previous studies [23,29,30,31,32]. A total of 5 items was used, measuring 2 types of online sexual activities dating back 6 months: Solitary OSAs (2 items; α = 0.939; e.g., “I watched sexually explicit material on the internet”) and Partnered OSAs (3 items; α = 0.813; e.g., “I have exchanged intimate photos with another person”). Each item was scored on a 6-point Likert scale (1 = not at all; 6 = almost daily), and the final scores were obtained by adding the corresponding items.

Attachment style. Attachment style was measured using The Revised Adult Attachment Scale (R-AAS) [33]. The scale consists of 18 items grouped into three attachment characteristics: Close (6 items; α = 0.728; e.g., “I find it relatively easy to get close to people”), Depend (6 items; α = 0.685; e.g., “I am comfortable depending on others”) and Anxiety (6 items; α = 893; e.g., “I often worry that other people don’t really love me”). Each item was scored on a 5-point Likert scale (1 = not at all characteristic of me; 5 = very characteristic of me). Attachment dimensions were obtained by calculating the additional subscale Avoidance, scored by summing the reversed items for subscales Close and Depend. The result indicates the extent to which a person has difficulties with closeness and dependence on others. According to the author’s instructions, attachment styles [34] were then assigned to each respondent along the dimensions Anxiety and Avoidance relative to the midpoint on the 5-point Likert scale: Secure (Anxiety < 3 and Avoidance < 3), Preoccupied (Anxiety > 3 and Avoidance < 3), Dismissive (Anxiety < 3 and Avoidance > 3) and Fearful (Anxiety > 3 and Avoidance > 3).

Sexual self. The model for the sexual self-concept used in this study was proposed by Deutsch [5] and contains six factors: Arousal, Exploration, Anxiety, Sexual Self-esteem and Sexual Self-efficacy. Sexual Arousal was measured using 9 items (α = 0.893; e.g.,”I get easily aroused”), Sexual Exploration was measured using 9 items (α = 0.849; e.g., “I would like to experiment when it comes to sex”), Sexual Anxiety was measured using 10 items (α = 0.783; e.g., “It would be difficult for me to relax during sex”), Sexual Self-esteem was measured using 15 items (α = 0.890; e.g., “I trust people find me attractive”) and Sexual Self-efficacy was measured using 13 items (α = 0.819; e.g., “Refuse to do something sexual with your partner that makes you uncomfortable”). Each item was scored on a 5-point Likert scale (1 = totally disagree; 5 = totally agree) except items for Sexual Self-efficacy, which were measured on a 6-point Likert scale (0 = not at all confident; 5 = very confident). The final scores were obtained by adding the corresponding items. 

Sexual satisfaction. Sexual satisfaction was measured with The New Sexual Satisfaction Scale-Short Form (NSS-S) [35]. This scale consists of 12 items that measure 2 aspects of sexual satisfaction, Ego-focused (6 items; α = 0.926; e.g., “The way I react sexually to my partner”) and Partner and Activity-focused (6 items; α = 0.910; e.g., “My partners’ letting go during sex”). The final scores were obtained by adding the corresponding items.

### 2.3. Data Analysis

Statistical analyses were performed using JASP (v. 0.13.1) [36] and IBM SPSS statistics (v. 26.0.0.0) [37]. First, cases with missing values and inconsistent responses were removed from the analysis, thus reducing the sample size to *n* = 244. Next, in order to identify subgroups within our sample, a cluster analysis was performed based on the sexual self-model we employed (Arousal, Anxiety, Openness, Sexual Self-esteem and Self-efficacy). This was a two-step process in which two clustering methods were employed. First, a hierarchical cluster analysis was employed using Ward’s method (D2) and Euclidian distance measure, followed by a K-means analysis used to maximize homogeneity within clusters and maximize heterogeneity between clusters. The stability of the cluster structure obtained with the two methods was evaluated using Cramer’s V test. Cluster profiling and external correlates (frequency of app use, motives for app use, attachment dimensions, online sexual activities, sexual satisfaction and number of sexual partners in the last 12 months) were examined using analysis of variance (ANOVA) with post hoc tests (Tukey and Games–Howell). In addition, log-linear analysis was used to test the association between categorical data (gender, relationship status, cyber infidelity and attachment style) and cluster membership with χ^2^ post hoc tests.

## 3. Results

### 3.1. Cluster Analyses

**Figure 1:**
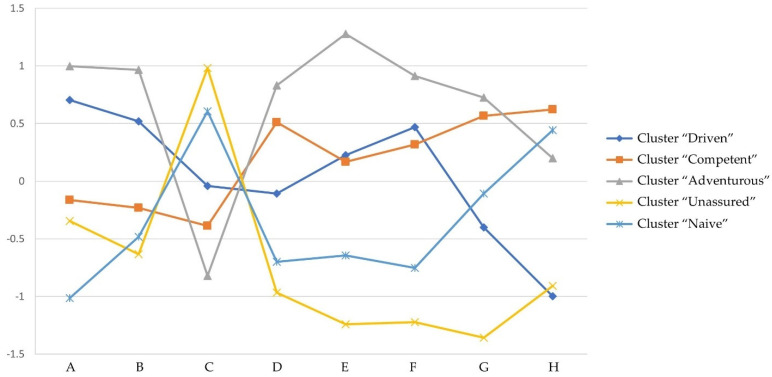
Cluster profiles by standardized mean values of Sexual Self components. A = Sexual Arousal; B = Sexual Exploration; C = Sexual Anxiety; D = Sexual Self-Esteem: Attractiveness; E = Sexual Self-Esteem: Behavior; F = Sexual Self-Esteem: Conduct; G = Sexual Self-Efficacy: Assertive; H = Sexual Self-Efficacy: Resistive.

**Figure 2:**
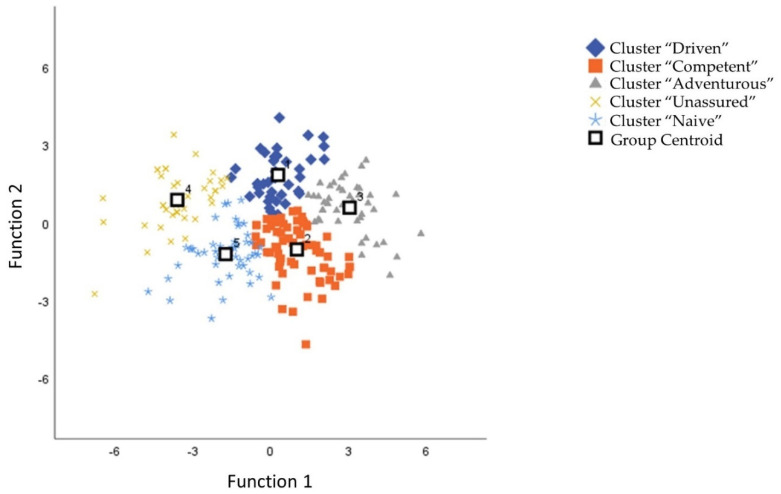
Five-cluster solution plotted in discriminant function space.

All variables included in the analysis were standardized to share the same metric. First, a hierarchical cluster analysis was performed using Ward’s method (D2) and the Euclidian distance measure. Visual inspection of the resulting dendrogram indicated a possible five-cluster solution. For cluster and membership validation, an additional K-means analysis was performed. Next, the agreement between the two methods was evaluated using the Chi-square test and Cramer’s V effect size measurement. Results of the analysis indicated a strong correspondence between cluster memberships as assigned by the two methods (*Cramer’s V* = 0.771, *ρ* < 0.001), which indicated that similar clusters were obtained regardless of the method used to derive them. Additionally, the chi-square test showed strong confidence in this result (χ^2^ (16) = 579.85, *ρ* < 0.001). In order to further confirm separation among clusters, the scores for each variable included in the cluster analysis (components of the sexual self-concept) were introduced into a discriminant function analysis, grouped by the identified clusters. The final cluster sizes exceeded 10% of the sample as recommended by Hair et al. [38]. The profiles of the four clusters are presented in Figure 1, and the cluster plot is depicted in Figure 2.

### 3.2. Cluster Description

Descriptive statistics and results of the ANOVA tests for the variables included in the cluster analysis are shown in Table 1.

The first cluster, labeled “Driven”, included individuals with moderate levels of sexual self-esteem. They felt more confident about the way they conduct themselves in sexual situations, but they were less assured in regards to their physical appearance and performance in the sexual domain. Individuals in this cluster showed a particular configuration that indicated a certain lack in the capacity for self-inhibition in the sexual area. High arousal and interest in sexual variety and exploration were coupled with a significantly low score on the ability to refuse sexual advances or restrain their own sexual impulses. Anxiety levels were moderate, indicating a certain feeling of uneasiness relating to sexual encounters. This group consisted of 40 individuals (16.4% of the total sample), mostly males (70%), and had the largest percentage of sexually active (90%) and committed (72.5%) individuals. It also had the highest percentage of infidelity (12.5%) (all males). A small percentage of this group declared themselves bisexual (10%) and homosexual (2.5%), with the majority being heterosexual (87.5%).

The second cluster, labeled “Competent”, included individuals with high scores in the area of sexual self-esteem, reporting confidence in their physical attractiveness and feeling comfortable with their ability to perform sexually and conduct themselves in sexual situations. Additionally, they had the highest sexual self-efficacy scores, meaning that they are confident in their ability to communicate their sexual desires and refuse unwanted sex. Sexual arousal and interest in sexual exploration were moderate, with intermediate levels between the other groups. Individuals in this cluster reported feeling only somewhat anxious about sex. This was the largest group (*n* = 73, 29.9% of the total sample), and it consisted mostly of females (79.5%). A high percentage of individuals were sexually active (75.3%), most of them being committed to a partner (58.9%). A small percentage of this group declared themselves bisexual (9.5%), with the majority being heterosexual (90.5%).

The third cluster, labeled “Adventurous”, was characterized by the highest levels of sexual self-esteem and a moderate level of self-efficacy. Individuals in this group reported feeling extremely confident with their sex appeal and sexual performance and also with their ability to assert their sexual needs. However, the capacity for self-restraint and refusal of sexual advances was intermediate between the other clusters. Sexual arousal, disinhibition and interest in exploring sexual options were at the highest levels in this cluster. Additionally, individuals in this cluster reported feeling almost no anxiety regarding sexual activities. This group consisted of 45 individuals (18.4% of the total sample), with an even gender distribution (51% female; 49% male). It had the second-largest percentage of sexually active individuals (80%) and infidelity (8.88%) (all males), and 64.5% of members were in a committed relationship. A small percentage of this group declared themselves bisexual (8.8%) and homosexual (4.4%), with the majority being heterosexual (86.6%).

The fourth cluster, labeled “Unassured”, consisted of individuals with particularly low levels of sexual self-esteem and extremely high sexual anxiety. Their sexual-self configuration indicates substantial dissatisfaction with their physical appearance, sexual performance and the way they conduct themselves in sexual situations. In addition, members of this cluster declared having serious difficulties in expressing their sexual desires, managing unwanted sexual advances or refusing to engage in sexual behaviors. Nevertheless, they exhibited a low-to-moderate level of sexual arousal and some interest in sexual exploration, although to a lesser extent than members of the other clusters. Sexual anxiety had the highest levels in this cluster, indicating that sexual matters pose significant difficulties associated with an incapacity to relax during intercourse, discomfort and feelings of shame and guilt. This group consisted of 39 individuals (15.9% of the total sample), mostly female (61.5%), with 64.1% being sexually active and 56.4% being in a committed relationship. A small percentage of this group declared themselves bisexual (10.2%), with the majority being heterosexual (89.7%).

The final cluster, labeled “Naive”, included individuals with low levels of sexual self-esteem, indicating some dissatisfaction with their appearance and sexual performance and a lack of confidence in their ability to conduct themselves in sexual situations. Additionally, they reported feeling somewhat able to assert their sexual desires, but they were very confident in their capacity to refuse sexual advances. Members of this group had the lowest reported levels of sexual arousal and declared themselves less interested in experimenting sexually and rather more sexually inhibited. Sexual anxiety levels were high, meaning that sex was associated with tenseness, discomfort and feelings of guilt and shame. This group consisted of 47 individuals (19.2% of the total sample), mostly females (68%). Compared to the cluster “Driven”, the majority of individuals were not sexually active (55.3%), and only 38.3% of members were in a committed relationship. A small percentage of this group declared themselves bisexual (14.8%) and homosexual (4.2%), with the majority being heterosexual (80.8%). 

### 3.3. Cluster Comparisons

First, the clusters were compared based on variables relating to app use (see Table 2). Results of the ANOVA indicated no statistically significant differences between groups regarding frequencies of app use. Nevertheless, inspection of cluster means revealed that the clusters “Driven” and “Adventurous” showed the highest Tinder and Instagram use frequencies. Regarding motives for app use, there were no significant mean differences between clusters for the motives Love, Ease of communication, Self-worth validation and Trend. However, Clusters “Driven” and “Adventurous” had the highest mean values for Casual Sex and Thrill of Excitement motives, while cluster “Naïve” had the lowest values.

Next, the clusters were compared based on variables measuring certain aspects of sexuality: OSAs, satisfaction and number of sex partners in the last 12 months (see Table 2). Regarding sexual satisfaction, clusters “Competent” and “Adventurous” reported the highest levels, followed by clusters “Driven”, “Unassured” and “Naïve”. Not surprisingly, the number of sex partners in the previous 12 months was significantly higher for clusters “Driven” and “Adventurous”, followed by clusters “Competent”, “Unassured” and “Naïve”. In addition, the highest engagement in OSAs, both solitary and partnered, was reported by sexually “Driven” and “Adventurous” individuals. The cluster “Competent” had the lowest mean score for solitary OSAs, while the cluster “Unassured” had the lowest mean score for partnered OSAs.

Finally, the groups were compared based on attachment orientation (see Table 2). The two-way log-linear analysis examining the association between cluster membership and attachment style showed that the interaction cluster by attachment style (χ^2^ (12) = 52.398, ρ < 0.0001) was significant. In order to evaluate this effect, χ^2^ post hoc tests were performed separately for each cluster. Results indicated that subjects with secure attachment were more represented in clusters “Competent” (χ^2^ (1) = 15.13, ρ < 0.001) and “Adventurous” (χ^2^ (1) = 6.71, ρ < 0.01) and less represented in clusters “Unassured” (χ^2^ (1) = 9.86, ρ < 0.01) and “Naïve” (χ^2^ (1) = 14.59, ρ < 0.002). In contrast, subjects with fearful attachment were less represented in clusters “Competent” (χ^2^ (1) = 8.49, ρ < 0.01) and “Adventurous” (χ^2^ (1) = 8.94, ρ < 0.01) and more represented in the cluster “Unassured” (χ^2^ (1) = 21.07, ρ < 0.000). In regards to attachment orientation, sexually “Competent” and “Adventurous” groups exhibited the lowest mean levels of attachment anxiety and avoidance, while the sexually “Unassured” and “Naïve” groups reported the highest levels. 

The analysis of the demographic data (see Table 3) showed no significant effect based on age. The two-way log-linear analysis examining the associations between cluster membership, gender, relationship status, seeking a partner online and cyber infidelity showed that the interactions cluster by gender (χ^2^ (4) = 29.986, ρ < 0.0001), cluster by relationship status (χ^2^ (8) = 27.615, ρ < 0.001) and cluster by cyber infidelity (χ^2^ (4) = 33.74, ρ < 0.001), were statistically significant, while the interaction cluster by seek partner online (χ^2^ (4) = 7.557, ρ = 0.109) was not significant in the model. In order to evaluate these effects, χ^2^ post hoc tests were performed separately for each cluster. Results indicated that males were overrepresented in the cluster “Driven” (χ^2^ (1) = 19.45, ρ < 0.001) and underrepresented in the cluster “Competent” (χ^2^ (1) = 14.82, ρ < 0.001). Gender was not significantly associated with clusters “Adventurous”, “Unassured” and “Naïve”. Regarding relationship status, subjects in a relationship were less represented in the cluster “Naïve” (χ^2^ (1) = 9.06, ρ < 0.001), while sexually inactive single subjects were less represented in the cluster “Driven” (χ^2^ (1) = 8.47, ρ < 0.001) and overrepresented in the cluster “Naïve” (χ^2^ (1) = 19.36, ρ < 0.001). No other post hoc test reached statistical significance. As for cyber infidelity, subjects were more represented in clusters “Driven” (χ^2^ (1) = 14.29, ρ < 0.001) and “Adventurous” (χ^2^ (1) = 11.02, ρ < 0.001). Although the interaction cluster by seeking a partner online failed to reach significance, frequency analysis results showed a preference to seek partners online, especially in cluster “Driven”. In contrast, the cluster “Naïve” had the lowest percentage of subjects that used the online environment to look for a potential partner.

## 4. Discussion

The aim of this study was to examine characteristics of online app use within specific configurations of the sexual self-concept. In order to pursue this objective, we determined if reliable sexual-self subtypes can be identified among individuals. Similar to the findings of Buzwell and Rosenthal [10], five clusters were identified, each representing specific configurations concerning sexual drives, sexual anxiety, sexual self-esteem and sexual self-efficacy. Variables related to app use, attachment and sexuality were then examined within this sexual self-concept framework in an attempt to further expand knowledge on what differentiates people in their online behaviors and activities. This research adds to the existing literature on sexuality and the online environment by employing a profile-based approach in investigating specificities of the interaction between individuals and the digital social environment.

### 4.1. Contribution

This study contributes to research regarding sexuality and the digital environment by offering an additional framework for understanding what drives people in their app use and related sexual outcomes. 

Consistent with previous research [12], we found that individuals in our sample with intense sexual desire and interest in exploring sexual options were more likely to use apps, such as Tinder, in order to seek romantic or sexual experiences. Additionally, they reported the highest variety of sexual partners and a greater frequency of engagement in online sexual activities. Conversely, those who reported lower levels of openness to sexual experiences were more interested in using apps for social approval or online interactions rather than for sexual purposes and were less likely to use Tinder or engage in online sexual activities. We did not find any evidence of problematic online sexual activities, as the relatively low rates of engagement are not indicative of problematic behavior and might be more related to recreational purposes [32,39,40].

These results were evidenced by the cluster comparisons where sexually “Driven” and “Adventurous” individuals reported the highest frequencies of Tinder use and online sexual activities, greater variety of sex partners, more interest in using apps for sexual experiences and higher rates of cyber infidelity. Although similar in regards to their sexual outcomes, the dynamics that drive their behavior in the online romantic scene seem to be quite different. While the sexually “Adventurous” profile denotes strong agreement between the sexual self-components, the sexually “Driven” style does not seem to be fully coherent in its configuration. High sexual desire and need for sexual exploration come in conflict with their relative uneasiness about sex, doubts about their sex appeal and performance and low capacity to refuse sex. This particular combination might link sexuality to efforts in self-esteem regulation or to fear of rejection, as shown by the tendency of these individuals to use apps for social approval and because they feel more comfortable in online interactions. The high levels of attachment anxiety reported in this group supports this hypothesis, as it has been associated previously with more short-term sexual encounters, hypersexuality [41], high numbers of extradyadic relationships [42] or with engaging in sex as a mean to reduce insecurities, establish closeness, prevent rejection or boost self-esteem [43]. On the other hand, sexually “Adventurous” individuals did not seem conflicted in their sense of themselves as sexual beings. Their high confidence in their ability to assert themselves sexually coupled with their heightened interest in the sexuality domain allows them to navigate the online social and dating scene much more freely in the pursuit of pleasure and new experiences, as evidenced by their lack of interest in using apps for self-worth validation or for feeling more comfortable in online interactions. In our sample, this group reported low levels of attachment anxiety and avoidance, a result that adds to the existing body of conflicting evidence regarding insecure attachment and casual sex [43,44,45,46,47]. Overall, for these two groups, sexual interest seemed to play an important role in what motivates their app use, particularly Tinder. It could simply be that Tinder offers a suitable platform for those interested in access to many potential mates that one would otherwise not have a chance to meet and date, thus increasing the probability of serial encounters and sexual intercourse. This situation is extremely appealing to individuals with a high sex drive, as it provides multiple opportunities to gratify their needs for novelty and sexual experiences. The sexual drive of “Adventurous” and “Driven” individuals was also translated into online sexual activities, with these groups reporting greater frequencies of engagement in both solitary and partnered sexual activities, such as watching pornography or sex chat with strangers, regardless of their relationship status. Results in this study are not sufficient to determine whether there is an association, for these groups, between particular apps used and various sexual outcomes, online or offline. Future studies could be aimed at investigating individuals with these sexual profiles in more detail in order to gain a more complex picture regarding their relation to social apps and sexual behaviors and also to track possible developments in their sense of sexual self.

In contrast, individuals not driven by their sexual urges reported significantly lower interest in using apps for sexual encounters, less Tinder use, less variety of sexual partners and engagement in online sexual activities. However, they were more likely than sexually “Driven” and “Adventurous” individuals to use apps because they felt better relatedness to others online rather than offline and for social approval. This result could be linked to lower levels of sexual self-esteem and more sexual anxiety. For these groups, particularly the sexually “Unassured” and “Naïve”, future research could be focused on the impact of the online environment, more specifically social/dating apps and online sexual interactions, on their sexual self-concept.

The lowest interest in using Tinder was found among sexually “Competent” individuals, who exhibited moderate sexual preoccupations, solid awareness of their sexual needs and felt in control of their sexuality. They reported being very satisfied with their sex life while having fewer sex partners than sexually “Driven” and “Adventurous” individuals. This suggests more conservative attitudes towards promiscuity and the capacity to sustain more stable romantic relationships. Given their sexual profile and predominantly secure attachment orientation, people with this sexual style did not revolve around sexual urges in their app use and seemed more able to balance their expectations regarding the online romantic and sexual scene. Similarly, sexually “Naïve” individuals were more inclined to use apps for romantic purposes, but, overall, they were not highly motivated in their app use and showed little interest in dating apps, like Tinder. This is hardly surprising, given that Tinder, although initially introduced as a dating app, is often associated with the “hook-up” culture and generally seen as a sex app. In their study of sexual self-profiles, Buzwell and Rosenthal [10] described this group as “presexual” with little to no sexual experience and holding abstract notions of sexuality. In line with these descriptions, we found that this group had the highest percentage of sexually inactive individuals and the lowest percentage of committed individuals. However, the frequency of engagement in online sexual activities was not significantly different from the other moderate-to-low sex drive clusters, which could indicate a positive development in their sexuality associated with the online environment. On the other hand, their propensity to refuse sex might interfere with the need to secure attachment objects, a situation in which consenting to a sexual activity, online or offline, would have little to do with expanding awareness about their sexual self. In comparison, sexually “Unassured” individuals, despite having a negative perception of themselves as sexual agents, were more likely to use Tinder, suggesting some interest in engaging the online dating scene and exploring their sexual drives. Nevertheless, these efforts did not necessarily translate into sexual outcomes despite the high reported levels of insecure attachment. This could be explained by the fact that this group reported the highest levels of sexual anxiety and very little confidence in their ability to express sexual needs and feel in control in sexual situations. For both “Unassured” and “Naïve” individuals, anxious attachment needs did not associate with more sexual encounters, as they reported experiencing high sexual anxiety and low sexual self-esteem. It could be that what is missing in the research of attachment and sexuality is the analysis of sexual styles, as in our study, we found evidence that both supports and disputes previous results on this topic.

In conclusion, our findings suggest that people’s perceptions of themselves as sexual beings play an important role in determining why and how they engage the online dating, social and sexual scenes. This approach could offer new directions for future research and a framework for better understanding online social interactions and their sexual outcomes by taking into consideration not only sexual self-components but also the personal meaning that people attribute to their sense of sexual self. Uncovering individual sexual dynamics and understanding how they guide the people in their online romantic and sexual endeavors could have the potential to inform app developers to better tailor their platform concepts for a better fit with the needs of specific populations and help promote more healthy environments. Additionally, in regards to attachment theory, our results suggest that the sexual self-concept could play an important role in determining sexual outcomes related to attachment orientations.

### 4.2. Limitations

A number of limitations in the present study should be noted. First, the cross-sectional approach we used in this study does not allow for conclusions regarding causality or the circular effects that the variables may induce [48]. A longitudinal research design should offer the opportunity for better insight on dynamics concerning the sexual self and the online environment. Another issue concerns the potential bias in the data given the use of self-reported measures [49,50]. Second, the results of this research were obtained within the specific cultural and economic landscape of Romania. Hence, comparison with other populations from different cultures and with different values is needed in order to explore the general or particular nature of these results [51]. Third, given the lack of consensus in the research field on how the sexual self-concept should be defined and measured, the use of only one model may fail to capture relevant dimensions of this construct. In addition, future studies with larger sample sizes could be aimed at better understanding how experiences in the online environment relate to perceptions of oneself as a sexual and relational being and the way in which this impacts the individual and society at large. Finally, the small sample size used in this study does not allow for definite conclusions about the relations between the studied variables, and results should be interpreted with caution. Increased resolution is needed for a better understanding of sexual profiles and their particularities and utility in understanding online behavior.

## 5. Conclusions

In summary, we replicated previous findings concerning sexual self-profiles, and we also analyzed these profiles in relation to motives for app use, in particular Tinder, which emerged as the most utilized dating app in our sample. Next, we investigated differences among these groups based on attachment orientation and sexuality-related measures. To our knowledge, this is the first study attempting to profile app users based on sexual self-characteristics.

We found that sexual self-characteristics can be reliably grouped into five different sexual styles, in accordance with Buzwell and Rosenthal’s [10] proposed model. Individuals in these groups reported some notable differences in their app use, sexual outcomes and attachment styles, indicating that the sexual self-concept might be an important factor in social and dating app use as well as in sexually related online activities. However, the relatively small size of our sample forces a cautious interpretation of the results, and therefore, we recommend future replication with larger sample sizes.

## Figures and Tables

**Table 1:** Cluster profiles.

Variables	1. Driven	2. Competent	3. Adventurous	4. Unassured	5. Naive
	*n* = 40	*n* = 73	*n* = 45	*n* = 39	*n* = 47
	M (SD)	M (SD)	M (SD)	M (SD)	M (SD)
Arousal	35.8 (4.99) ^2,4,5^	29.5 (5.24) ^1,3,5^	37.93 (4.05) ^2,4,5^	28.17 (5.73) ^1,3,5^	23.3 (5.9) ^1,2,3,4^
Exploration	34.57 (5.71) ^2,4,5^	29.02 (5.55) ^1,3^	37.88 (5.14) ^2,4,5^	26.02 (7.31) ^2,3^	25.5 (7.83) ^1,3^
Anxiety	19.37 (4.99) ^3,4,5^	17.26 (4.07) ^3,4,5^	14.62 (3.57) ^1,2,4,5^	25.61 (6.82) ^1,2,3^	23.31 (4.5) ^1,2,3^
Self-esteem					
Attractiveness	21.45 (3.62) ^2,3,4,5^	24.06 (2.15) ^1,4,5^	25.42 (3.36) ^1,4,5^	17.8 (3.94) ^1,2,3^	18.9 (3.14) ^1,2,3^
Behavior	20.9 (1.75) ^3,4,5^	20.71 (1.77) ^3,4,5^	24.28 (0.99) ^1,2,4,5^	16.17 (3.04) ^1,2,3,5^	18.1 (1.8) ^1,2,3,4^
Conduct	16.85 (2.02) ^3,4,5^	16.41 (1.84) ^3,4,5^	18.15 (1.58) ^1,2,4,5^	11.87 (1.97) ^1,2,3,5^	13.2 (2.3) ^1,2,3,4^
Self-efficacy					
Assertive	26.42 (3.73) ^2,3,4^	20.3 (2.53) ^1,4,5^	20.93 (2.58) ^1,4,5^	12.59 (2.71) ^1,2,3,5^	17.6 (2.55) ^2,3,4^
Resistive	21.85 (5.78) ^2,3,5^	32.87 (4.18) ^1,3,4^	30 (5.11) ^1,2,4^	22.46 (6.63) ^2,3,5^	31.6 (3.9) ^1,4^

Note. Tukey and Games–Howell post hoc tests were both used, given that homogeneity of variance was not present in all cases and the clusters had unequal sizes. Indices represent cluster numbers and denote significant comparisons (ANOVA).

**Table 2:** Cluster comparisons.

Variables	1. Driven	2. Competent	3. Adventurous	4. Unassured	5. Naïve
	*n* = 40	*n* = 73	*n* = 45	*n* = 39	*n* = 47
	M (SD)	M (SD)	M (SD)	M (SD)	M (SD)
Frequency of app use					
Tinder	2.4 (1.9)	1.76 (1.29)	2.17 (1.76)	2.02 (1.58)	1.8 (1.32)
Instagram	4 (2.33)	3.72 (2.05)	3.86 (2.1)	3.59 (2.04)	3.31 (2.18)
App Motives					
Love	15.95 (5.46)	15.82 (5.31)	16.8 (5.66)	16.17 (5.38)	15.19 (5.53)
Casual sex	11.22 (4.2) ^2,4,5^	8.74 (4.14) ^1,3^	11.86 (4.77) ^2,4,5^	8.28 (3.99) ^1,3^	8.21 (3.58) ^1,3^
Ease of communication	14.62 (5.27)	14.05 (5.26)	12.8 (4.23)	14.48 (4.29)	13.91 (4.47)
Self-worth	14.17 (4.74)	13.27 (5.51)	12.91 (6.31)	14.17 (5.03)	12.29 (5.59)
Thrill of excitement	6.12 (2.11) ^5^	5.26 (2.14)	5.84 (2.4) ^5^	5.76 (1.85)	4.59 (1.97) ^1,3^
Trend	8.17 (3.09)	8.05 (3.09)	8.2 (3.88)	7.76 (2.55)	6.95 (2.75)
No. of sex partners	2.4 (2.93) ^4,5^	1.47 (1.41) ^4,5^	2.04 (2.54) ^4,5^	0.84 (0.74) ^1,2,3^	0.7 (0.6) ^1,2,3^
OSAs					
Solitary	10.72 (3.4) ^2,4,5^	7.52 (3.32) ^1,3^	11.31 (3.19) ^2,4,5^	7.56 (4.06) ^1,3^	8.08 (3.53) ^1,3^
Partnered	5.4 (2.63) ^4^	4.72 (2.08)	5.75 (2.96) ^4^	3.87 (1.67) ^1,3^	4.17 (2.63)
Sexual satisfaction					
Ego-focused	24.3 (3.5) ^3,4,5^	24.15 (4.39) ^3,4,5^	26.64 (4.08) ^1,2,4,5^	19.2 (5.76) ^1,2,3^	19.2 (6.6) ^1,2,3^
Partner-focused	21.3 (4.69) ^5^	23.5 (5.27) ^4,5^	24.44 (5.25) ^4,5^	18.76 (5.86) ^2,3^	17.8 (6.1) ^1,2,3^
Attachment					
Anxiety	3.06 (0.92)	2.63 (1.09) ^4^	2.45 (0.98) ^4,5^	3.52 (1.08) ^3^	3.08 (1.06) ^3^
Avoidance	2.88 (0.54)	2.73 (0.58) ^4,5^	2.78 (0.6) ^4^	3.22 (0.59) ^2,3^	3.03 (0.61) ^2^
Style (%)					
Secure	22.5	56.1 **	57.8 **	15.4 **	21.2 **
Preoccupied	30	15	11.1	10.2	23.4
Fearful	30	13.7 **	11.1 **	56.5 **	34
Dismissive	17.5	15	20	17.9	21.2

Note. Tukey and Games–Howell post hoc tests were used, given that homogeneity of variance was not present in all cases and the clusters had unequal sizes. Indices represent cluster numbers and denote significant comparisons (ANOVA). ** ρ < 0.01.

**Table 3:** Demographics.

Variables	1. Driven	2. Competent	3. Adventurous	4. Unassured	5. Naïve
	*n* = 40	*n* = 73	*n* = 45	*n* = 39	*n* = 47
	M (SD)	M (SD)	M (SD)	M (SD)	M (SD)
Age	27.22 (9.24)	25.54 (9.26)	27.02 (8.81)	28.66 (10.71)	27.25 (8.83)
(%) Gender M/F	70/30 **	20.5/79.5 **	49/51	38.5/61.5	32/68
(%) Relationship					
Single—inactive	10 **	24.6	20	35.9	55.3 **
Single—active	17.5	16.4	15.5	7.7	6.4
In a relationship	72.5	58.9	64.5	56.4	38.3 **
(%) Seeking online	72.5	60.2	53.3	58.9	44.6
(%) Cyber infidelity	27.5 **	4.1	24.4 **	0	2.1

Note. ** ρ < 0.01.

## Data Availability

Results and dataset can be made available by request.

## References

[1] Valkenburg P.M., Peter J. (2011). Online Communication Among Adolescents: An Integrated Model of Its Attraction, Opportunities, and Risks. J. Adolesc. Health.

[2] Petre C.E. (2021). The relationship between Internet use and self-concept clarity: A systematic review and meta-analysis. Cyberpsychol. J. Psychosoc. Res. Cyberspace.

[3] Bergman L.R., Magnusson D. (1991). Stability and change in patterns of extrinsic adjustment problems. Problems and Methods in Longitudinal Research: Stability and Change.

[4] Frost D.M., McClelland S.I., Clark J.B., Boylan E.A. (2014). Phenomenological Research Methods in the Psychological Study of Sexuality.

[5] Deutsch A.R., Hoffman L., Wilcox B.L. (2013). Sexual Self-Concept: Testing a Hypothetical Model for Men and Women. J. Sex Res..

[6] Kimmel M.S. (2007). The Sexual Self: The Construction of Sexual Scripts.

[7] Rostosky S.S., Dekhtyar O., Cupp P.K., Anderman E.M. (2008). Sexual Self-Concept and Sexual Self-Efficacy in Adolescents: A Possible Clue to Promoting Sexual Health?. J. Sex Res..

[8] Antičević V., Jokić-Begić N., Britvić D. (2017). Sexual self-concept, sexual satisfaction, and attachment among single and coupled individuals. Pers. Relatsh..

[9] Potki R., Ziaei T., Faramarzi M., Moosazadeh M., Shahhosseini Z. (2017). Bio-psycho-social factors affecting sexual self-concept: A systematic review. Electron. Physician.

[10] Buzwell S., Rosenthal D. (1996). Constructing a sexual self: Adolescents’ sexual self-perceptions and sexual risk-taking. J. Res. Adolesc..

[11] Aboujaoude E. (2012). Virtually You: The Dangerous Powers of the E-Personality.

[12] Sumter S.R., Vandenbosch L. (2018). Dating gone mobile: Demographic and personality-based correlates of using smartphone-based dating applications among emerging adults. New Media Soc..

[13] Barrada J., Castro Á. (2020). Tinder Users: Sociodemographic, Psychological, and Psychosexual Characteristics. Int. J. Environ. Res. Public Health.

[14] Rochat L., Bianchi-Demicheli F., Aboujaoude E., Khazaal Y. (2019). The psychology of “swiping”: A cluster analysis of the mobile dating app Tinder. J. Behav. Addict..

[15] Sevi B. (2019). The Dark Side of Tinder. J. Individ. Differ..

[16] Ciocca G., Robilotta A., Fontanesi L., Sansone A., D’Antuono L., Limoncin E., Nimbi F., Simonelli C., Di Lorenzo G., Siracusano A. (2020). Sexological Aspects Related to Tinder Use: A Comprehensive Review of the Literature. Sex. Med. Rev..

[17] Cooper A., Morahan-Martin J., Mathy R.M., Maheu M. (2002). Toward an Increased Understanding of User Demographics in Online Sexual Activities. J. Sex Marital. Ther..

[18] Shaughnessy K., Byers E.S., Walsh L. (2010). Online Sexual Activity Experience of Heterosexual Students: Gender Similarities and Differences. Arch. Sex. Behav..

[19] Mitchell K.J., Finkelhor D., Wolak J. (2003). The Exposure of Youth to Unwanted Sexual Material on the Internet: A National Survey of Risk, Impact, and Prevention. Youth Soc..

[20] Peter J., Valkenburg P.M. (2008). Adolescents’ Exposure to Sexually Explicit Internet Material and Sexual Preoccupancy: A Three-Wave Panel Study. Media Psychol..

[21] Koletić G. (2017). Longitudinal associations between the use of sexually explicit material and adolescents’ attitudes and behaviors: A narrative review of studies. J. Adolesc..

[22] Boies S.C., Cooper A., Osborne C.S. (2004). Variations in Internet-Related Problems and Psychosocial Functioning in Online Sexual Activities: Implications for Social and Sexual Development of Young Adults. Cyberpsychol. Behav..

[23] Wéry A., Canale N., Bell C., Duvivier B., Billieux J. (2020). Problematic online sexual activities in men: The role of self-esteem, loneliness, and social anxiety. Hum. Behav. Emerg. Technol..

[24] Blais-Lecours S., Vaillancourt-Morel M.-P., Sabourin S., Godbout N. (2016). Cyberpornography: Time Use, Perceived Addiction, Sexual Functioning, and Sexual Satisfaction. Cyberpsychol. Behav. Soc. Netw..

[25] Gutiérrez-Puertas L., Hernández V.M., Gutierrez-Puertas V., Granados-Gámez G., Rodríguez-García M.C., Aguilera-Manrique G. (2019). Online sexual activities among university students: Relationship with sexual satisfaction. An. Psicol./Ann. Psychol..

[26] Shaughnessy K., Byers E.S., Clowater S.L., Kalinowski A. (2013). Self-Appraisals of Arousal-Oriented Online Sexual Activities in University and Community Samples. Arch. Sex. Behav..

[27] McKenna K.Y.A., Green A.S., Smith P. (2001). Demarginalizing the sexual self. J. Sex Res..

[28] Sumter S., Vandenbosch L., Ligtenberg L. (2017). Love me Tinder: Untangling emerging adults’ motivations for using the dating application Tinder. Telemat. Inform..

[29] Byers E.S., Shaughnessy K. (2014). Attitudes toward online sexual activities. Cyberpsychol. J. Psychosoc. Res. Cyberspace.

[30] Wéry A., Billieux J. (2016). Online sexual activities: An exploratory study of problematic and non-problematic usage patterns in a sample of men. Comput. Hum. Behav..

[31] Döring N., Mohseni M.R. (2018). Are Online Sexual Activities and Sexting Good for Adults’ Sexual Well-Being? Results From a National Online Survey. Int. J. Sex. Health.

[32] Liu Y., Yue C., Zheng L. (2019). Influence of online sexual activity (OSA) perceptions on OSA experiences among individuals in committed relationships: Perceived risk and perceived infidelity. Sex. Relatsh. Ther..

[33] Collins N.L. (1996). Revised Adult Attachment Scale. Behav. Ther..

[34] Bartholomew K., Horowitz L.M. (1991). Attachment styles among young adults: A test of a four-category model. J. Personal. Soc. Psychol..

[35] Stulhofer A., Buško V., Brouillard P., Fisher T.D., Davis C.M., Yarber W.L., Davis S.L. (2011). The New Sexual Satisfaction Scale and its short form. Handbook of Sexuality-Related Measures.

[36] JASP Team (2020). JASP (Version 0.14.1). Computer Software..

[37] IBM Corp (2019). IBM SPSS Statistics for Windows.

[38] Hair J.F., Black W.C., Babin B.J., Anderson R.E. (2010). Multivariate Data Analysis: A Global Perspective.

[39] Grov C., Gillespie B.J., Royce T., Lever J. (2010). Perceived Consequences of Casual Online Sexual Activities on Heterosexual Relationships: A U.S. Online Survey. Arch. Sex. Behav..

[40] Ross M.W., Månsson S.-A., Daneback K. (2011). Prevalence, Severity, and Correlates of Problematic Sexual Internet Use in Swedish Men and Women. Arch. Sex. Behav..

[41] Ciocca G., Pelligrini F., Mollaioli D., Limoncin E., Sansone A., Colonnello E., Jannini E.A., Fontanesi L. (2021). Hypersexual behavior and attachment styles in a non-clinical sample: The mediation role of depression and post-traumatic stress symptoms. J. Affect. Disord..

[42] Jore J., Green B., Adams K., Carnes P. (2016). Attachment Dysfunction and Relationship Preoccupation. Sex. Addict. Compuls..

[43] Stefanou C., McCabe M.P. (2012). Adult Attachment and Sexual Functioning: A Review of Past Research. J. Sex. Med..

[44] Cooper M.L., Pioli M., Levitt A., Talley A.E., Micheas L., Collins N.L., Mikulincer M., Goodman G.S. (2006). Attachment styles, sex motives, and sexual behavior. Dynamics of Romantic Love: Attachment, Caregiving, and Sex.

[45] Davis D., Shaver P.R., Widaman K.F., Vernon M.L., Follette W.C., Beitz K., Davis D., Shaver P.R., Widaman K.F., Vernon M.L. (2006). “I can’t get no satisfaction”: Insecure attachment, inhibited sexual communication, and sexual dissatisfaction. Pers. Relatsh..

[46] Goldsmith K.M., Dunkley C.R., Dang S.S., Gorzalka B.B. (2016). Sexuality and romantic relationships: Investigating the relation between attachment style and sexual satisfaction. Sex. Relatsh. Ther..

[47] Roșca A.C., Mateizer A., Enea V. (2021). Tulburările de atașament. Abordarea Psihologică a Adopției și Asistenței Maternale. Polirom.

[48] Dan C.-I., Roşca A.C., Mateizer A. (2020). Job Crafting and Performance in Firefighters: The Role of Work Meaning and Work Engagement. Front. Psychol..

[49] Roșca A.C., Ciuraru A.C. (2017). The Influence of Emotional Intelligence on Anxiety and Defense Mechanisms of People Diagnosed with Glaucoma. Rom. J. Exp. Appl. Psychol..

[50] Roșca A., Burtăverde V., Dan C.-I., Mateizer A., Petrancu C., Iriza A., Ene C. (2021). The Dark Triad Traits of Firefighters and Risk-Taking at Work. The Mediating Role of Altruism, Honesty, and Courage. Int. J. Environ. Res. Public Health.

[51] Roșca A.C., Mateizer A., Dan C.-I., Demerouti E. (2021). Job Demands and Exhaustion in Firefighters: The Moderating Role of Work Meaning. A Cross-Sectional Study. Int. J. Environ. Res. Public Health.

